# Near-complete genome sequences of equine herpesvirus (EHV) 1, EHV-3, and EHV-4 from the USA using a hybrid assembly approach

**DOI:** 10.1128/mra.01347-25

**Published:** 2026-05-18

**Authors:** Tirth Uprety, Lutz S. Goehring, Aveen Rozhbayani, Allison Meyer, Stephanie Michelle Todd, Sahar Abdelrazek, Kevin Lahmers, Erdal Erol

**Affiliations:** 1 University of Kentucky Veterinary Diagnostic Laboratory, Lexington, Kentucky, USA; 2 Maxwell H. Gluck Equine Research Center, University of Kentucky 4530, Lexington, Kentucky, USA; 3 Department of Biomedical Sciences and Pathobiology, Virginia-Maryland College of Veterinary Medicine, Blacksburg, Virginia, USA; Katholieke Universiteit Leuven, Leuven, Belgium

**Keywords:** equine herpesvirus, hybrid assembly

## Abstract

We report near-complete genome sequences of equine herpesvirus (EHV) 1, 3, and 4 from the USA. Hybrid assemblies combining Illumina and Oxford Nanopore reads yielded high-quality genomes, enhancing understanding of EHV diversity and evolution in the USA.

## ANNOUNCEMENT

Of the nine equine herpesvirus (EHV) that are members of the family *Herpesviridae*, genus *Varicellovirus*, EHV-1, EHV-4, and EHV-3 are major pathogens of equids worldwide (1–6). There are limited sequences of EHV-4 and EHV-3 worldwide and none from the USA. Here, we report near-complete genome sequences of two EHV-4 isolates (KY22-1 and KY18UT29), one EHV-3 isolate (KY25-1), and seven EHV-1 isolates (KY17UT30, KY21UT70, KY23UT75, KY24UT16, KY24UT26, KY24UT38, and KY24UT61) (Table 1) using hybrid assembly of short (Illumina MiSeq) and long (Oxford Nanopore Technologies [ONT]) reads. Hybrid assembly has been used previously for human herpesviruses, helping overcome limitations of both short- and long-read technology (7–10).

**TABLE 1:** Clinical information, genome statistics, and sequencing data for EHV-1, EHV-3, and EHV-4 isolates

Isolate	GenBank ID	Genome length (bp)/GC %	Total number of reads/coverage (Illumina)	Total number of reads/coverage (Nanopore)	N50 (Nanopore)	Source/clinical history	Collection date (yr-mo)	Accession number (BLASTn %, closest match)
EHV-4 isolate KY22-1	PP765803	144,789/50.28	3,263,696/2,714×	70,793/113×	3,445	Nasal swab (respiratory infection)	2022-05	LC075587.1 (99.79)
EHV-4 isolate KY18UT29	PQ630624	144,617/50.26	971,166/391×	507,742/498×	2,544	Pooled tissue (abortion)	2018-12	LC063142.1 (99.70)
EHV-1 isolate KY17UT30	PQ630625	148,241/56.49	1,317,003/332×	309,184/160×	2,086	Nasal swab (respiratory infection)	2017-11	NC_001491.2 (99.93)
EHV-1 isolate KY21UT70	PQ630626	148,567/56.41	1,507,622/1,268×	19,735/35×	1,009	Pooled tissue (abortion)	2021-03	KT324731.1 (99.90)
EHV-1 isolate KY23UT75	PQ630627	150,226/56.66	1,474,282/1,227×	13,089/22×	731	Pooled tissue (abortion)	2023-02	NC_001491.2 (99.85)
EHV-1 isolate KY24UT16	PQ630628	149,680/56.52	986,844/353×	33,983/48×	1,750	Pooled tissue (abortion)	2024-01	LC109622.1 (99.90)
EHV-1 isolate KY24UT26	PQ630629	149,562/56.55	1,712,100/514×	35,169/44×	1,519	Pooled tissue (abortion)	2024-02	NC_001491.2 (99.86)
EHV-1 isolate KY24UT38	PQ630630	149,540/56.50	2,221,405/2,201×	23,759/39×	713	Pooled tissue (abortion)	2024-01	LC109622.1 (99.87)
EHV-1 isolate KY24UT61	PQ630631	149,221/56.57	863,873/591×	58,861/87×	4,175	Pooled tissue (respiratory infection)	2024-01	KT324731.1 (99.88)
EHV-3 isolate KY25-1	PV541150	151,555/68.09	285,019/106×	1,119,406/328×	998	Oral swab (oral ulceration)	2025-02	NC_024771.1 (99.79)

Nasal swabs, oral swabs, and pooled fetal tissues (Table 1) that were qPCR positive only for EHV-1, EHV-4, or EHV-3 were used for virus isolation. Equine dermal cells (EHV-4) or fetal equine kidney cells (EHV-1 and EHV-3) were used for isolation (11). All samples originated from Kentucky, USA. A culture supernatant (passage 0) was used for DNA extraction using the DNeasy Blood and Tissue Kit. This DNA was subjected to both short- and long-read sequencing. Illumina libraries were prepared using NexteraXT and sequenced on a MiSeq platform using MiSeq v2 or v3 chemistry, generating paired-end reads of 150, 250, or 300 bp. Nanopore libraries were prepared with the ONT Rapid Barcoding Kit (SQK-RBK114.24) and sequenced on MinION Mk1B devices using R10.4.1 flow cells. Base calling and demultiplexing were performed using Dorado (v7.4.13) within MinKnow (v24.06.8) employing the super accurate model. Samples were sequenced across multiple MiSeq and MinION runs.

Reads were further processed using fastp (v0.24.0) on a *Q* score of 10 and a minimum length of 100 for nanopore sequences (12) and a *Q* score of 30 and a minimum length of 30 for Illumina reads. Host-derived reads were removed by mapping to the *Equus caballus* reference genome (EquCab3.0) genome using Bowtie2 (v2.5.3) (13). Approximately 0.3%–75% of Illumina reads and 5%–88% of Nanopore reads mapped to the host genome and were removed prior to downstream analysis. Remaining reads were subjected to both *de novo* assembly and reference-guided assembly. *De novo* assemblies were generated using Flye (for Nanopore reads) and SPAdes. Viral contigs were identified by BLASTn analysis against the NCBI nucleotide database. For reference-guided consensus generation, reads were mapped to reference genomes LC075587 (EHV-4), NC_001491.2 (EHV-1), and NC_024771 (EHV-3) using Minimap2 (v2.28) (14), followed by consensus calling with Ococo (v0.1.2.6) (15). Assemblies were polished by mapping Illumina reads using BWA-MEM (v0.7.18) (16) and performing a single round of Pilon (v1.20) (17). Assemblies were annotated in Geneious Prime (v2024.0.4) using the transfer annotation tool. For EHV-4 isolate KY22-1, small unresolved terminal repeat regions in the short-read assembly were resolved using long-read consensus sequences. The assembled genomes showed >99.7% nucleotide identity to their closest corresponding GenBank reference strains. These genome sequences provide updated genomic data for EHV circulating in the USA and support future studies of viral evolution and pathogenicity.

## Data Availability

All sequencing data generated in this study have been deposited in the NCBI Sequence Read Archive (SRA) under BioProject PRJNA1354462. The Illumina and Oxford Nanopore reads for each genome are available under the following BioSamples and SRA accession numbers: PP765803 (SAMN52945520, SRR35896946, and SRR35896944), PQ630624 (SAMN52945521, SRR35896945, and SRR35896943), PQ630625 (SAMN52945522, SRR35896934, and SRR35896942), PQ630626 (SAMN52945523, SRR35896933, and SRR35896941), PQ630627 (SAMN52945524, SRR35896932, and SRR35896940), PQ630628 (SAMN52945525, SRR35896931, and SRR35896939), PQ630629 (SAMN52945526, SRR35896930, and SRR35896938), PQ630630 (SAMN52945527, SRR35896929, and SRR35896937), PQ630631 (SAMN52945528, SRR35896928, and SRR35896936), and PV541150 (SAMN52945529, SRR35896927, and SRR35896935).
